# The Influence of Prior Knowledge on Perception and Action: Relationships to Autistic Traits

**DOI:** 10.1007/s10803-016-2701-0

**Published:** 2016-01-28

**Authors:** Gavin Buckingham, Elizabeth Evgenia Michelakakis, Gnanathusharan Rajendran

**Affiliations:** Department of Psychology, Heriot-Watt University, David Brewster Building, Edinburgh, EH14 4AS UK

**Keywords:** Autistic quotient, Grip force, Object lifting, Size-weight illusion, Sensorimotor prediction

## Abstract

Autism is characterised by a range of perceptual and sensorimotor deficits, which might be related to abnormalities in how autistic individuals use prior knowledge. We investigated this proposition in a large non-clinical population in the context of the size-weight illusion, where individual’s expectations about object weight influence their perceptions of heaviness and fingertip forces. Although there was no relationship between autistic traits and the magnitude of the illusion, we observed an inverse relationship between AQ scores and how expectations influenced initial fingertip force application. These findings provide a novel dissociation between how perceptual and sensorimotor processes are related to autistic traits, and suggest that, autistic traits might explain some of the variance surrounding how individuals grip and lift objects.

## Introduction

Autism Spectrum Disorder (ASD) is a highly heritable and heterogeneous neurodevelopmental disorder. It is characterised by a variety of symptoms including language impairments, stereotyped behavioural patterns, and social difficulties (for recent review, see Jones et al. 2014). Currently, diagnosis is based upon a dyad of social interaction and communication skills, in addition to a restricted repertoire of interests and behaviours (DSM-V, APA, 2013). In recent years, high-level explanatory theories of ASD have moved from single deficit to multiple deficit accounts (see Rajendran and Mitchell 2007 for a review of the cognitive theories). In addition to differences at these high levels of cognition, an increasing body of work has indicated that ASD populations might exhibit atypical perception and deficits in sensorimotor control—a point acknowledged in the revised DSM-V criteria for ASD (DSM-V, APA 2013).

In terms of sensorimotor control, a range of studies have found evidence for movement deficits in populations with autism (for review, see Gowen and Hamilton 2013). A growing body of work suggests that the locus of many of these movement problems might stem from difficulties in utilizing prior information to guide feedforward control mechanisms. For example, Schmitz et al. (2003) compared postural responses of children with and without autism in a task where a mass they were holding was removed. Children without autism showed clear anticipatory postural adjustments, indicating that they were anticipating the removal of the load. Children with autism, by contrast, showed a much later postural response to having the load removed, suggesting that they were utilizing an online, feedback-driven control strategy in this task (see also Mosconi et al. 2015; Wang et al. 2014).

Although rarely considered as having specific perceptual impairments, a well-established body of work has demonstrated that ASD populations show atypical performance on a wide range of perceptual tasks. Most notably, studies have shown that ASD populations outperform matched control samples on embedded figure tasks and local versus global processing (Mitchell and Ropar 2004; Shah and Frith 1983). Interestingly, several studies have shown that ASD populations might be less affected by visual illusions which are based on an individual’s prior knowledge. Ropar and Mitchell (2002) examined the well-established perceptual bias to judge a slanted circle as appearing more circular than it actually is. This effect is typically taken to reflect ‘shape constancy’—the implicit integration of the prior knowledge that the shape is a circle with the input on the retina. The authors noted that ASD individuals were far less affected by their prior knowledge in conditions where other visual cues were removed, reporting the true shape of the slanted circle more accurately than control participants. Recently, Mitchell et al. (2010) have found a similar effect for the Shepard illusion, where the top surfaces of two identical, but differently-oriented, table surfaces appear to have markedly different aspect ratios from one another. This effect is typically understood as reflecting how individuals’ prior knowledge of depth cues, and their implicit assumption that tables have depth, influences their perception. The smaller illusory effect experienced by ASD individuals suggests that their conscious perception is not biased by prior knowledge as much as non-clinical populations.

Due to the heterogeneous nature of ASD, its many co-morbid disorders, and the difficulty these populations have with providing verbal reports, it has often been difficult to draw strong conclusions about the relationship between autism and perceptual/sensorimotor abnormalities. In recent years, to capitalise on the spectrum nature of the disorder, efforts have been made to classify non-clinical populations in terms of their autistic spectrum characteristics. The most popular of these scales, the Autism Spectrum Quotient (AQ), was developed by Baron-Cohen et al. (2001), and has been validated in a range of non-clinical contexts (e.g., Ruzich et al. 2015). This approach has already been successfully employed in a range of contexts (e.g., McKenna et al. 2015; Sutherland and Crewther 2010). Of particular relevance to the current work, Chouinard et al. (2013) examined the relationship between autistic traits and individuals’ susceptibility to three widely-studied visual illusions: the Müller-Lyer illusion, the Ponzo illusion, and the Ebbinghaus illusion. They reported an inverse correlation between AQ scores and the magnitude of the Müller-Lyer illusion (i.e., individuals with higher AQ scores experienced a less powerful illusion), but found no relationship between autistic traits and the magnitude of the Ponzo or Ebbinghaus illusions.

Although the role of prior knowledge in visual illusions has been well studied in ASD and its broader phenotype, no study has examined how autistic traits are reflected in perception and action within the same task. To this end, we examined fingertip force control and perceptions of heaviness in the context of the ‘Size-Weight Illusion’ (SWI), where prior knowledge influences perception and action in unique and dissociable ways. A large non-clinical sample of individuals lifted objects which induced the SWI, where small objects are judged as feeling heavier than larger objects of the same mass (Charpentier 1891). Typically, this illusion is taken to reflect how prior expectations, for example that large objects are likely to be heavier than small objects, are integrated with sensory input to form the conscious experience of the smaller objects feeling heavier than the larger objects (Buckingham 2014; Buckingham and Goodale 2010a; Flanagan et al. 2008). In contrast to the majority of other integration effects in perception (e.g., Ernst and Banks 2002), the experience of an object’s heaviness reflects the inverse of the standard Bayesian optimal integration between priors and sensory input (Brayanov and Smith 2010)—the objects which the lifter expects to feel light end up feeling heavier than they actually are, especially in comparison to heavier-looking counterparts of the same mass.

In the context of object lifting, prior expectations do not only influence perceptions of heaviness. Because of the feedforward, predictive, nature of how we grip and lift objects, the apparent heaviness of an object also influences the forces used to lift it. Therefore, novel heavy-looking objects are lifted with more force than novel light-looking objects—regardless of how much they actually weigh (Gordon et al. 1992). Thus, when interacting with objects which induce the SWI, individuals tend to lift the large (heavy-looking) object with more force than the small (light-looking) object. Interestingly these sensorimotor prediction ‘errors’ do not persist and, in stark contrast to the static and unchanging perceptual illusion, individuals rapidly adapt their fingertip force rates from the expected to the actual (and identical) weights of the illusion-inducing objects (Flanagan and Beltzner 2000). Thus, the level of gripping and lifting forces used to lift an object is dissociated from how heavy that object subsequently feels when its weight is judged (see also Buckingham and Goodale 2010b, 2013; Grandy and Westwood 2006).

To date, no study has systematically investigated what drives individual differences in the magnitude of these perceptual and sensorimotor effects in non-clinical populations. To this end, we examined how autistic traits were related to the perception of object weight, sensorimotor prediction, and fingertip force adaptation, in a classic SWI experiment. If a high prevalence of autistic traits does impact upon an individual’s ability to integrate prior knowledge into their conscious perceptual experience, it is likely that individuals with higher AQ scores will experience a reduced illusion in addition to making smaller size-induced prediction errors when they lift the large and small objects for the first time.

## Methods

### Participants

Eighty-five volunteers (37 female, mean age = 22.0 years, SD = 3.6) took part in a simple object lifting study. The majority of participants (75) were self-reported right handers. All procedures were approved by the ethics board at Heriot-Watt University, and informed consent was obtained from all individual participants included in the study. All participants were university undergraduate students or members of the surrounding community, and had no reported sensorimotor deficits. In addition, none of the participants has been formally diagnosed with autism.

## Materials

Prior to undertaking the object lifting task, participants completed a pencil-and-paper version of the adult Autistic Quotient (AQ: Baron-Cohen et al. 2001; Woodbury-Smith et al. 2005). The 50 item questionnaire contains self-statements covering five domains associated with ASD: communication and social skills, attention to detail, attention switching, imagination and rigidness of interests and behaviour. Example statements include “I find it hard to make new friends”, “I tend to notice details that others do not” and “It does not upset me when my daily routine is disturbed”. Each statement is responded to on a 4 point Likert scale. This allows participants to indicate whether, they “definitely agree”, “slightly agree”, “slightly disagree” or “definitely disagree” with the statements. The AQ is scored out of 50 points with higher scores reflecting higher levels of autistic traits. Although the AQ was not developed to be a diagnostic tool, a score of 26 or above has been reported to be potentially indicative of Asperger syndrome (Woodbury-Smith et al. 2005) and 32 or above reported to be indicative of “clinically significant” levels of autistic traits (Baron-Cohen et al. 2001). In our sample, AQ scores ranged from 2 to 32, and the mean AQ score was 15.2 (SD = 5.7).

**Fig. 1:**
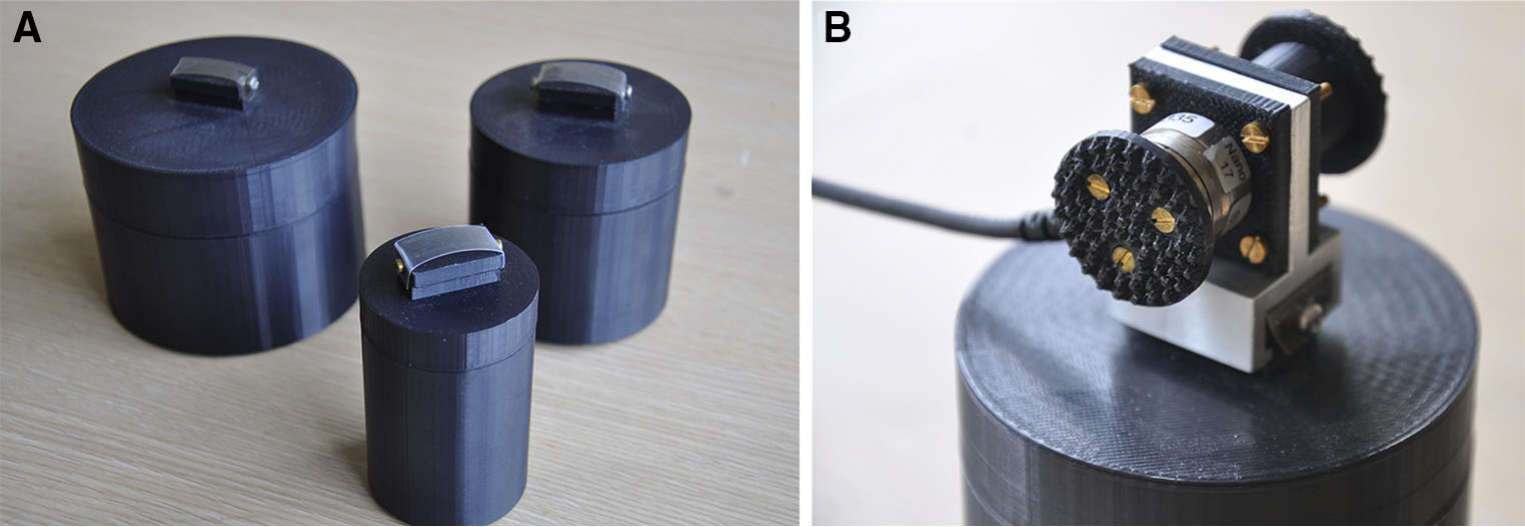
**a** The small, medium, and large cylinders lifted by participants and **b** the handle they used to grip and lift the cylinders

Participants lifted three homogenous black plastic cylinders with a constant height (7.5-cm) and mass (400-g), but varying diameters (Fig. 1a). The small cylinder had a diameter of 5-cm, the medium cylinder had a diameter of 7.5-cm, and the large cylinder had a diameter of 10-cm. Each cylinder had a mount attached to the centre of its top surface, which facilitated the rapid attachment of an aluminium and plastic handle containing an ATI Nano-17 force transducer (Fig. 1b), which recorded forces in 3 dimensions at 1000 Hz. During the experiment, participants wore LCD PLATO shutter goggles (Translucent Technologies) which occluded their vision in between lifts of the objects.

### Procedure and Analysis

After completing a pencil-and-paper version of the AQ, participants undertook the experimental trials. On each trial, participants were seated with their dominant hand resting on the table in front of them, with the lenses of the shutter goggles opaque. The experimenter then quietly placed one of the cylinders directly in front the participant. When the goggles opened, concurrent with an auditory cue, participants reached out with their dominant hand, grasped the handle attached to the top surface of the cylinder with their thumb and index finger, and lifted the object in a ‘smooth, controlled, and confident manner’. They were instructed to keep the object steady a few centimetres above the table surface until a second auditory cue 4-s after the start of the trial signalled them gently place the object back on the table’s surface. Participants then gave a numerical judgement about how heavy the object felt during the lift. There were no constraints on this scale, other than higher values would indicate a heavier-feeling object and vice versa (i.e., an arbitrary magnitude estimation—Zwislocki and Goodman 1980). Participants lifted each of the three objects 10 times in one of three pseudo-randomised orders, for a total of 30 lifts in a single session. Including the time taken to complete the AQ, the entire experiment lasted approximately 30 min.

The data extracted from the force transducers were smoothed with a 14-Hz Butterworth filter. We defined the forces perpendicular to the surface of the handle as grip force and the vector sum of the remaining forces as load force. These force profiles were differentiated with a 5-point central difference equation to yield their rates of change. The peak value of the grip force rate (pGFR) and load force rate (pLFR) on the initial lift of each cylinder provided an index of sensorimotor prediction. The force rates used to lift the small cylinder were subtracted from the force rates used to lift the large cylinder on the first lifts of these objects, to provide an index of how size cues influence fingertip forces (pGFRdiff and pLFRdiff). The heaviness ratings given on each trial by each participant were normalized to a z-distribution, and the average value given to each cylinder was taken to reflect their perceptions of object weight. The magnitude of the SWI on a particular trial was calculated as the rating given to the large cylinder subtracted from the rating given to the small cylinder.

## Results

### Effect of Object Size on Perceptions of Heaviness and Fingertip Force Application

Prior to our analyses, we excluded five participants as outliers, due to the fact that they had a SWI which was three standard deviations above or below the mean, or exhibited an effect of object size on their force rates which was three standard deviations above or below the mean.

The remaining sample of 80 individuals showed clear indications that their perceptions of heaviness across all trials, and their fingertip forces on the first lift of each object, were influenced by the size of the cylinders. When examining average perceptions of heaviness with a one way within-subject ANOVA, we observed a significant main effect of object size (F(1,79) = 1147.9, *p* < .001; ηp^2^ = .94). Post hoc t-tests confirmed that participants experienced a robust SWI, reporting that small cylinder felt heavier than medium-sized cylinder (t(78) = 29.5, *p* < .001) or the large cylinder (t(78) = 42.3, *p* < .001), with the large cylinder feeling less heavy than the medium-sized cylinder (t(78) = 22.9, *p* < .001).

**Fig. 2:**
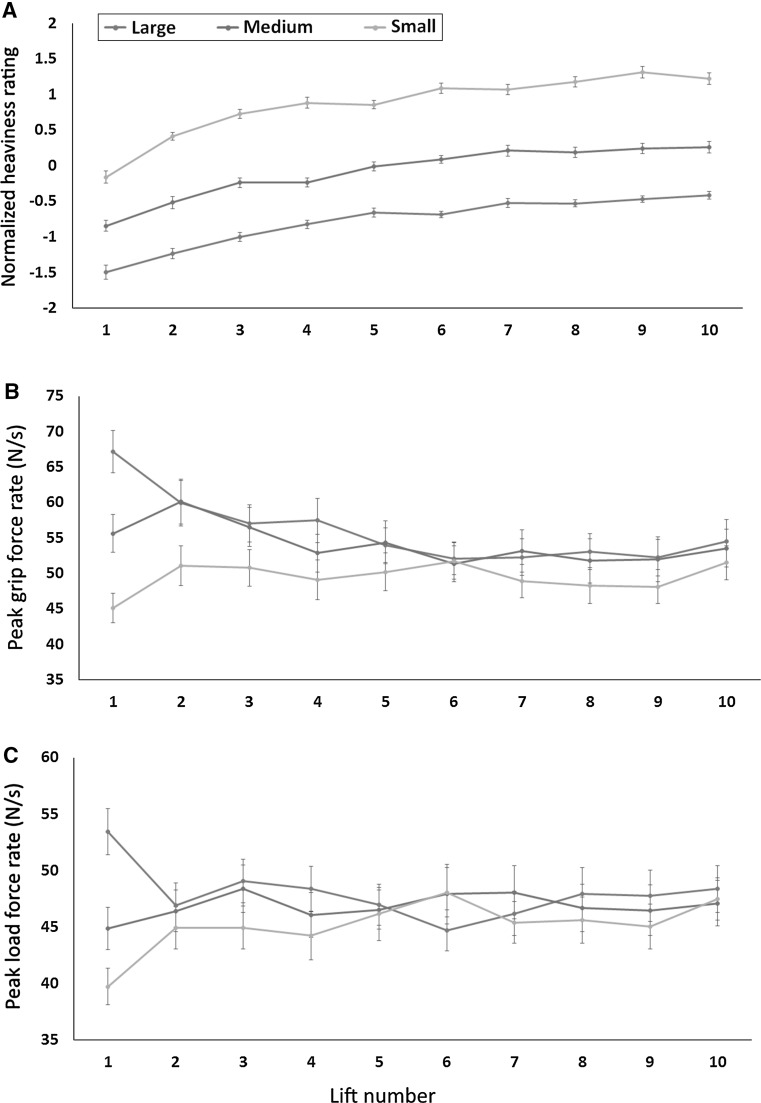
**a** The perceived heaviness ratings of each object across all trials, **b** the peak grip force rate (pGRF) applied to each object across all trials, and **c** the peak load force rate (pLFR) used to lift each object across all trials. *Error bars* indicate standard error of the mean

Similarly, we found a significant main effect of object size when examining the first-trial force rates for pGFR (F(1,79) = 84.1, *p* < .001; ηp^2^ = .32) and pLFR (F(1,79) = 48.8, *p* < .001; ηp^2^ = .26). Thus, on the first lift of each object, participants lifted the large cylinder with a higher rate force than the medium cylinder (pGFR: t(78) = 4.0, *p* < .001; pLFR: t(78) = 4.76, *p* < .001) or the small cylinder (pGFR: t(78) = 9.2, *p* < .001; pLFR: t(78) = 7.0, *p* < .001), and lifted the small cylinder with less force than the medium-sized cylinder (pGFR: t(78) = 4.4, *p* < .001; pLFR: t(78) = 2.8, *p* < .01). In short, our participants showed perceptual reporting (Fig. 2a) and sensorimotor prediction (Fig. 2b, c) that is consistent with numerous other studies using similar stimuli (Buckingham et al. 2012; Buckingham and Goodale 2010b; Flanagan and Beltzner 2000).

### Relationship Between Autistic Traits and Perception and Action During SWI

**Fig. 3:**
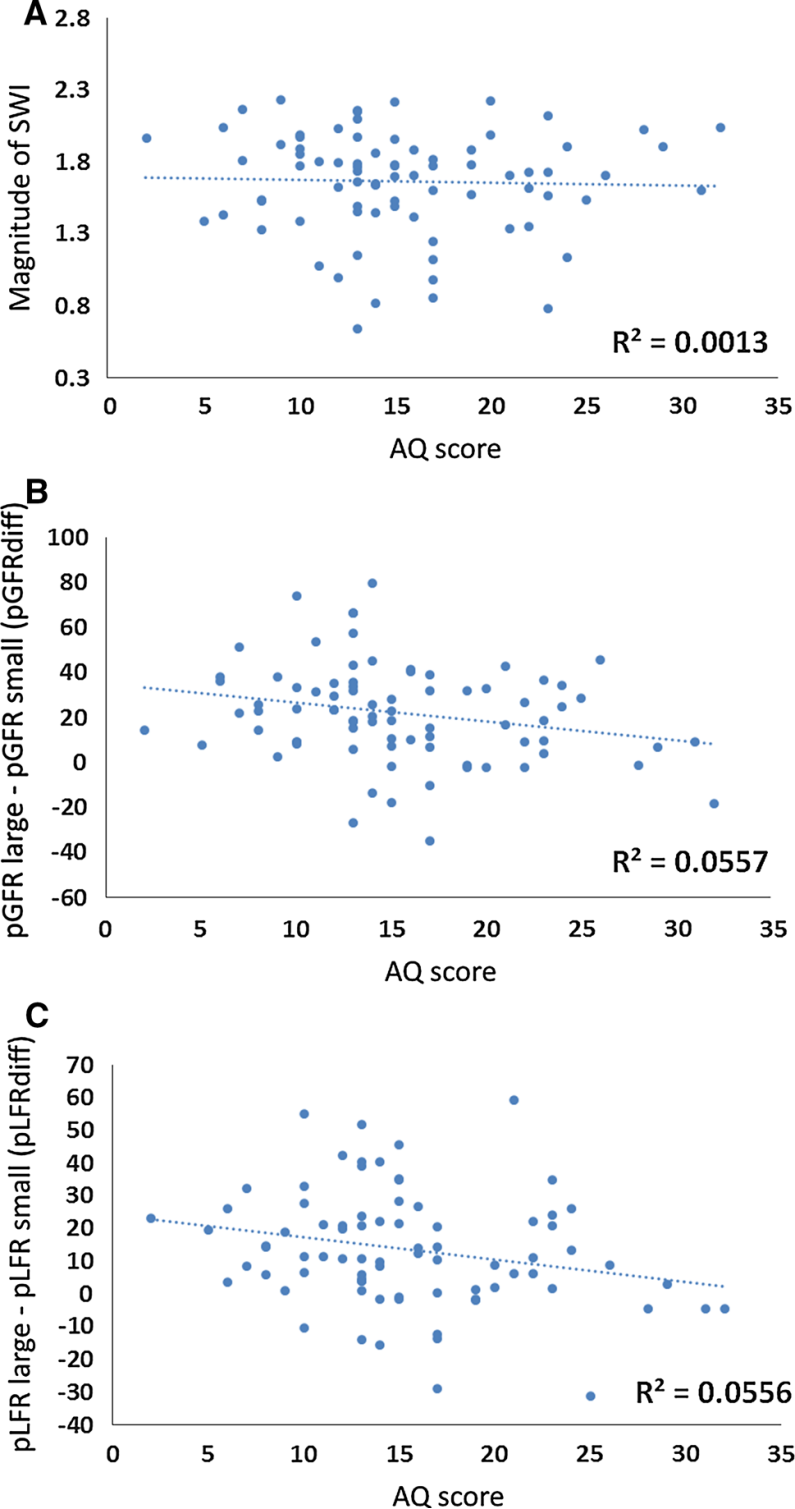
**a** Scatter plots highlighting the lack of relationship between the magnitude of the SWI and AQ scores, **b** the significant relationship between pGFRdiff and AQ scores, and **c** the significant relationship between pLFRdiff and AQ scores

To examine the relationship between autistic traits and the factors outlined above, we calculated a simple metric of the magnitude of the SWI across all trials by subtracting the average reported weight of the light-feeling large object from the average reported weight of the heavy-feeling small object. We calculated an analogous metric to quantify the magnitude of the effect of object size on the initial fingertip force rates by subtracting the pGFR and pLFR used to lift the small object from the pGFR and pLFR used to lift the large object. This index of how object size influences sensorimotor prediction is hereafter referred to as pGFRdiff and pLFRdiff. For direct comparison to these single-trial difference scores, we also calculated the magnitude of the SWI on trial 1 alone. We then examined the relationship between these metrics and individuals’ AQ scores in three separate Pearson’s correlations. Counter to our predictions, we observed no relationship between the magnitude of the SWI and AQ scores (r = −0.04, n = 80, *p* = .75; see Fig. 3a). Similarly, we observed no relationship between AQ scores and the SWI on the first trial in isolation (r = 0.13, n = 80, *p* = .27). There were, however, significant negative correlations between individuals’ AQ scores and pGFRdiff (r = −0.24 n = 80, *p* < .05; see Fig. 3b) as well as their pLFRdiff (r = −0.24, n = 80, *p* < .05; see Fig. 3c).

In order to determine whether the relationship between the size effect on force rates and autistic traits may be driven by other demographic variables which may be correlated with AQ scores, we performed separate stepwise linear regressions to explain the degree to which age, handedness, and gender were associated with pGFRdiff and pLFRdiff over and above individuals’ AQ scores. Of all of these factors, the AQ scores explained the most variance in both the pGFRdiff and pLFRdiff analyses (R^2^ = .056, *p* < .05 in both cases). No other variable explained enough variance to be included in the final model (all *p* values >.3).

We also examined the degree to which rates of fingertip force adaptation (i.e., trial-by-trial learning) was related to autistic traits. To this end, we calculated the unsigned difference between the force rate used to lift each object on the 2nd lift and the force rate used to lift each object on the 1st lift (see Fig. 2b, c). With this novel metric, higher values would indicate higher rates of learning from one trial to the next. Here, however, we found no significant relationship between rates of learning and autistic traits in terms of pGFR (large: r = .06, n = 80, *p* = .61; medium: r = −.17, n = 80, *p* = .12; small: r = .010, n = 80, *p* = .38) or pLFR (large: r = .−005, n = 80, *p* = 97; medium: r = −.18, n = 80, *p* = .11; small: r = .20, n = 80, *p* = .07). Thus, the relationship between action parameters and autistic traits appears to be limited to initial sensorimotor prediction, with no obvious effect on trial-by-trial learning.

## Discussion

This study examined if autistic traits influence the degree to which prior expectations affect perception and action. To this end, we measured the fingertip forces in a large non-clinical population while they lifted and judged the weight of objects which induce the SWI—cylinders which varied in volume, but had the same mass as one another. Our sample, on average, experienced a robust SWI, reporting that the small cylinder felt heavier than the medium-sized cylinder, which they in turn judged as feeling heavier than the large cylinder (Fig. 2a). The magnitude of this perpetual illusion did not, however, have any relationship to AQ scores in our sample (Fig. 3a), suggesting that the degree to which prior knowledge influences an individual’s perception of object weight is unrelated to their autistic traits.

These findings may indicate that the SWI does not, in fact, reflect how prior expectations are integrated into perceptual reports, instead being a consequence of a perceptual mechanism to detect variables other than object mass, such as density (Chouinard et al. 2009; Grandy and Westwood 2006), inertia tensor (Amazeen and Turvey 1996), or throwability (Zhu and Bingham 2011). However, given that it has been directly shown that expectations contribute at least partially to the experience of the SWI (Buckingham and Goodale 2010a; Flanagan et al. 2008), we feel it more likely that our results indicate that the ability to integrate past perceptual experiences is *not* related to autism features in our non-clinical sample. This finding is surprising given earlier work showing that autistic populations, in contrast to non-autistic controls, do not integrate prior knowledge about objects into their visual experience of object shape (e.g. Ropar and Mitchell 2002). The results from the current work suggest that autistic populations would experience just as robust a SWI as other non-clinical populations—a conclusion that is particularly surprising given recent findings showing that ASD populations have a range of impairments in their processing of tactile stimuli (Puts et al. 2014), and in the integration of visual-tactile information (Poole et al. 2015a, b).

Our other main finding from this dataset was related to the forces which participants used to lift the objects for the first time. Our participants showed the classic pattern of behaviour with these novel stimuli—lifting the large cylinder with a higher rate of grip and load force than the small cylinder on trial 1 (Fig. 2b, c). This behaviour is typically taken to reflect an individual’s sensorimotor expectations that large objects will outweigh small objects, driven by the consistent positive correlation between size and mass encountered in the real world (Gordon et al. 1991). Here—in contrast to our SWI data—we found an inverse relationship between participants’ sensorimotor expectations and their AQ scores (Fig. 3b, c), indicating that individuals with more autistic traits are less inclined to incorporate past information into their motor programmes when interacting with novel objects. Follow-up regression analyses confirmed that this relationship was not driven by other correlated factors, such as participants’ gender. This finding might represent a novel form of a motor deficit, suggesting that individuals with ASD might make less accurate/efficient sensorimotor predictions when interacting with objects in the real world where, on average, predictive behaviour is likely to be advantageous. It is worth noting that there we found no relationship between autistic traits and the other fingertip force measure described in this study—the rate of trial-to-trial learning, suggesting that this an effect specific to the use of prior information, rather than a generalized sensorimotor issue. These results are the first, to our knowledge, to show an aspect of sensorimotor control related to autistic traits in a non-clinical population, highlighting the potential sensitivity of this measure. Our findings are also in line with recent studies showing reduced metrics of predictive control in different object lifting paradigms in ASD populations (Mosconi et al. 2015; Schmitz et al. 2003; Wang et al. 2014). It remains an open question, however, about the degree to which other cues that have been shown to influence sensorimotor prediction, such as material cues and arbitrary learned associations (Buckingham et al. 2009; Chouinard et al. 2005), are related to autistic traits.

Our findings suggest that the sensorimotor aspects of the ASD phenotype requires greater emphasis—both in understanding ASD, but also in potentially providing an endophenotype (Gottesman and Gould 2003; Iarocci et al. 2007) and/or a biomarker via the broader autism phenotype. The DSM-V (APA 2013) has re-highlighted the need to understand the sensorimotor aspects of ASD. Indeed, some have argued that ASD needs to be viewed in its totality as both a perceptual-motor and social cognitive disorder (McKenna et al. 2015; Mostofsky and Ewen 2011; Rajendran and Mitchell 2007). As a counterpoint to the dominance of the social-cognitive aspects of ASD, it may be that some of the social-communication consequences of ASD may be due to more primary differences in perception–action developmental. Indeed, Mostofsky and Ewen (2011) argue that social and communicative competence depends on the development of skilled behaviours and that these skilled behaviours reside in the brain as internal action models (Shadmehr and Mussa-Ivaldi 1994). Consequently, the autism phenotype may therefore arise from the anomalous formation of internal action models, as has been argued by Mostofsky and Ewen (2011) who propose that internal action models are used in a “feedforward” fashion to extrapolate and understand the actions of others for theory of mind (Baron-Cohen et al. 1985; Klin et al. 2003).

Further, the exact mechanism of how ‘top-down’ (prior knowledge) effects influences individuals with ASD, and its broader phenotype, is as yet unknown. We consider our findings in the context of the general autism phenotype, which includes both individuals with ASD and those without a clinical diagnosis. However, although we exercise caution in extrapolating too far AQ results into clinical samples (see also Gregory and Plaisted-Grant 2013), we suggest that this research is a starting point to see if our findings will not only be replicated in a clinical sample, but also if the magnitude of these effects will be reflected in differences that we might expect between a clinical sample and an AQ sample. Future work will compare the current measures of sensorimotor prediction between ASD and control populations, to confirm (1) whether this effect is more prevalent in clinical populations and (2) whether this failure to integrate prior expectations might underpin the widely-debated range of different sensorimotor deficits in ASD individuals. This measure is particularly appealing as a potential marker of ASD, not only in terms of sensorimotor training, but also for the purposes of early diagnosis. The object lifting task outlined in the current work is simple to administer, and the influence of size cues on sensorimotor prediction has been found in children as young as 3 years of age (Gordon et al. 1992), suggesting it is a robust behaviour which can be examined early in development. Perhaps most importantly, the tendency to lift heavy-looking objects with more force is a completely implicit behaviour which does not rely on verbal reporting or participant motivation – two factors which are difficult to disentangle from traditional perceptual and sensorimotor measures (Fisk and Goodale 1989; Raymond and O’Brien 2009).

To sum up, the current work examined how autistic traits are related to perception and action in the context of the size-weight illusion. In a large sample of neurotypical individuals, we found no relationship between AQ scores and the magnitude of the perceptual effect. We did, however, find a small, but significant, relationship between AQ scores and sensorimotor prediction, both in terms of grip and load force rates. These findings point towards a potential dissociation of processes with perception unaffected, but with action affected, by autistic traits. Future work should examine this paradigm in clinically-diagnosed ASD samples to determine the possible efficacy for this test as a biomarker and endophenotype for ASD.
